# Assessment of internal refractive index profile of stochastically inhomogeneous nuclear models via analysis of two-dimensional optical scattering patterns

**DOI:** 10.1117/1.JBO.26.5.055001

**Published:** 2021-05-10

**Authors:** Dizem Arifler, Martial Guillaud

**Affiliations:** a Middle East Technical University, Northern Cyprus Campus, Physics Group, Kalkanli, Turkey; b British Columbia Cancer Research Center, Department of Integrative Oncology, Imaging Unit, Vancouver BC, Canada

**Keywords:** cell analysis, diagnostics, finite-difference time-domain modeling, Gaussian random fields, Haralick features, light scattering

## Abstract

**Significance:** Optical scattering signals obtained from tissue constituents contain a wealth of structural information. Conventional intensity features, however, are mostly dictated by the overall morphology and mean refractive index of these constituents, making it very difficult to exclusively sense internal refractive index fluctuations.

**Aim:** We perform a systematic analysis to elucidate how changes in internal refractive index profile of cell nuclei can best be detected via optical scattering.

**Approach:** We construct stochastically inhomogeneous nuclear models and numerically simulate their azimuth-resolved scattering patterns. We then process these two-dimensional patterns with the goal of identifying features that directly point to subnuclear structure.

**Results:** Azimuth-dependent intensity variations over the side scattering range provide significant insights into subnuclear refractive index profile. A particular feature we refer to as contrast ratio is observed to be highly sensitive to the length scale and extent of refractive index fluctuations; further, this feature is not susceptible to changes in the overall size and mean refractive index of nuclei, thereby allowing for selective tracking of subnuclear structure that can be linked to chromatin distribution.

**Conclusions:** Our analysis will potentially pave the way for scattering-based assessment of chromatin reorganization that is considered to be a key hallmark of precancer progression.

## 1 Introduction

Optical scattering signals obtained from tissues can provide significant information regarding the biological state of tissue constituents. This has been highly exploited in development of a series of scattering-based strategies to tackle various diagnostic challenges. Some notable efforts include assessment of abnormalities in morphological, structural, and biochemical properties of red blood cells in diseases such as malaria or anemia,^1^^–^^4^ optical oximetry at the capillary level to assess local tissue oxygenation and metabolic function,^5^ detection of virus infections,^6^ dynamical monitoring of apoptosis,^7^ identification and quantification of cellular or subcellular changes associated with precancer progression or field carcinogenesis,^8^^–^^12^ and testing for the presence of circulating tumor cell clusters which can have strong metastatic potential.^13^

Studies aimed at quantifying the extent of diagnostic contrast inherent in optical scattering signals play a key role in implementation and optimization of all the above-mentioned efforts. The level of complexity of tissue constituents, however, renders such studies far from trivial. In most cases, detailed numerical research needs to be carried out to establish links between disease-related alterations in these constituents and the resulting changes in their scattering properties. This, in turn, allows for proper interpretation of optical signals and paves the way for informed diagnostic decisions.

Numerical research has indeed contributed significantly to our understanding of how precancerous changes in cells affect their scattering signatures. A lot of progress in this respect can be attributed to our ability to construct increasingly realistic cell models and to the flexibility provided by computational techniques in simulating the optical response of these models. In epithelial tissues, the presence of large and irregularly shaped cell nuclei with increased DNA content and coarse chromatin distribution is generally considered to be a key indicator of precancer progression. Numerical studies suggest that changes associated with the overall morphology of nuclei lead to elevated small-angle scattering, whereas changes in internal refractive index profile due to chromatin reorganization lead to elevated high-angle scattering.^14^^–^^16^ It is hence evident that different angular ranges can potentially highlight different aspects of precancer progression.

Most numerical studies on optical scattering from tissue constituents focus on computation of scattered light intensity only in terms of the polar angle by averaging over the azimuthal direction. There is sound evidence, however, that azimuthally resolved scattering measurements aid in accurate determination of the size and shape of scatterers or offer additional insights into complex structures containing refractive index heterogeneities at different length scales.^17^^–^^29^ As a matter of fact, we previously performed an investigation of optical scattering from three-dimensional (3D) models of normal and precancerous epithelial cell nuclei as a function of the polar and azimuthal scattering angles; the results indicate that analysis of the degree of azimuthal asymmetry in two-dimensional (2D) intensity distributions can lead to identification of well-performing diagnostic metrics.^30^

In our prior numerical algorithms, each nuclear model was represented as an ellipsoid and an inhomogeneous profile was generated by inserting refractive index heterogeneities at different length scales. These heterogeneities were in the form of small ellipsoids of varying sizes and refractive indices placed randomly throughout the nucleus. Although this discrete approach resulted in construction of 3D models that were in agreement with the basic trends observed in histopathology images of normal and precancerous nuclei, it precluded a full characterization of the stochastic aspect of refractive index fluctuations. Alternatively, nuclei can be modeled as structures having a continuously varying refractive index distribution that conforms to a statistical correlation function. Such a stochastic approach has recently been widely adopted as a means of modeling cells or tissues^9^^,^^11^^,^^31^^–^^37^ and is expected to better capture the degree of inhomogeneity that characterizes normal and precancerous cell nuclei.^38^

In fact, changes in the degree of internal inhomogeneity of cell nuclei can be directly linked to chromatin reorganization that is considered to be a key hallmark of precancer progression. Conventional intensity features obtained from optical scattering signals are mostly dictated by the overall morphology and mean refractive index of nuclei. Hence, scattering-based assessment of nuclear inhomogeneity calls for further numerical research targeted toward identification of alternative features that can be used to selectively monitor internal refractive index fluctuations. In this study, we carry out a systematic investigation to elucidate how changes in internal refractive index profile of epithelial cell nuclei can best be detected via optical scattering. We first construct stochastically inhomogeneous nuclear models based on typical correlation lengths of subnuclear refractive index fluctuations as quantified from quantitative histopathology images of cervical tissue. We then numerically simulate the 2D scattering response of these models and identify features that provide information on their internal structure. The results presented demonstrate that analysis of azimuthal dependence in 2D scattering signals is essential for tracking subnuclear structure and offer significant insights into the potential of scattering-based assessment of chromatin distribution for diagnostic purposes. More generally, our work is expected to shed new light on the possibility of optical detection of any pathological condition that manifests as changes in refractive index profile of tissue constituents.

## 2 Methods

### 2.1 Analysis of Quantitative Histopathology Images

To fully characterize the refractive index profile of cell nuclei, we analyzed quantitative histopathology images of cervical biopsy samples sliced to 4-μm-thick sections and stained with Feulgen-thionin. All the samples were obtained and imaged at the British Columbia Cancer Research Center (Vancouver, Canada). The imaging system employed an illumination wavelength of 600 nm and a 20× objective with a numerical aperture of 0.75, yielding a resolution of 0.34  μm. Since Feulgen-thionin is stoichiometric for DNA, the optical density of a given image pixel is directly related to DNA concentration at that location.^39^ It is also well established empirically that refractive index depends linearly on concentration.^40^^,^^41^ These images can hence be processed to extract information on subnuclear refractive index fluctuations which can be linked to chromatin organization.

We first used a special software^39^ to carry out nuclear segmentation for each biopsy image. Next, we implemented a MATLAB^®^ (MathWorks, Inc., Natick, Massachusetts) routine to analyze segmented nuclear images and to extract the correlation length characterizing each nucleus. The refractive index profile of a nucleus was assumed to follow a Gaussian correlation function given as

$$C(r)=e^{-r^{2}/l_{c}^{2}},$$ (1)

where r is the distance between two points and $l_{c}$ is the correlation length which can be roughly defined as the length scale over which the correlation drops to a negligible level. Our routine computed the autocorrelation of optical density values for each row and column of a manually selected region of interest (ROI) in a given nuclear image. Gaussian functions in Eq. (1) were then fitted to autocorrelation values to extract the correlation length for each row and column of the selected ROI; averaging over all the fits with a minimum of four data points and a goodness-of-fit statistic of $R^{2}>0.95$ gave the correlation length for that ROI. This was repeated for three different ROIs for each nuclear image and the average was considered to be a representative value for the characteristic correlation length $l_{c}$ of the nucleus.

**Fig. 1:**
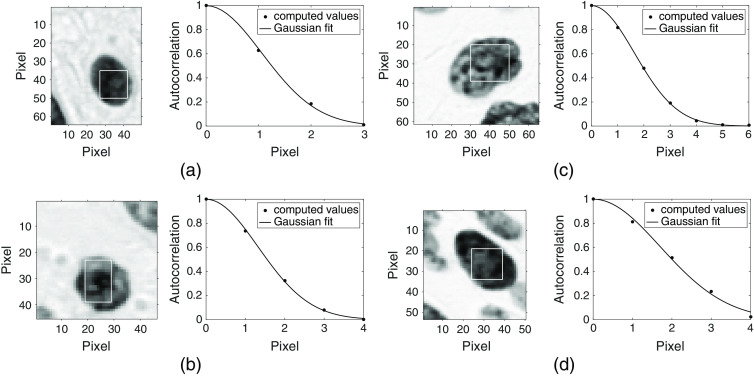
Nuclear images along with sample Gaussian fits to the autocorrelation of optical density values corresponding to a single row or column of a selected ROI. Note that the images are scaled differently, but the effective pixel sampling size is $0.34\times 0.34\;\mu m^{2}$ in all cases. The diagnostic categories and the extracted correlation lengths of the nuclei shown are: (a) negative, $l_{c}=1.79\text{ pixels}$ or 0.61  μm, (b) CIN 1, $l_{c}=2.05\text{ pixels}$ or 0.70  μm, (c) CIN 2, $l_{c}=2.73\text{ pixels}$ or 0.93  μm, and (d) CIN 3, $l_{c}=2.43\text{ pixels}$ or 0.83  μm.

We applied our routine to images of nuclei segmented from 10 biopsy pairs. These pairs consisted of a biopsy diagnosed with cervical intraepithelial neoplasia (CIN) and a negative biopsy obtained from the same patient. Figure 1 shows four different nuclear images along with sample Gaussian fits to the autocorrelation values corresponding to a single row or column of a selected ROI for each image. Note that the fits justify the use of Gaussian correlation functions to describe the spatial correlation of subnuclear refractive index fluctuations in cervical epithelium. Overall, our analysis of a total of 937 nuclei (including 476 negative nuclei, 155 CIN 1 nuclei, 168 CIN 2 nuclei, and 138 CIN 3 nuclei) revealed that the extracted correlation lengths ranged approximately between 0.4 and 1.0  μm. We hence employed this range in our simulations so as to span typical length scales of refractive index fluctuations in cervical cell nuclei.

### 2.2 Construction of Nuclear Models

#### 2.2.1 Spherical nuclear models that are optically denser than the embedding cytoplasm

Each nuclear model was constructed as a sphere of radius R and mean refractive index n embedded in a cytoplasmic medium with a refractive index of $n_{\text{out}}<n$. A stochastically inhomogeneous subnuclear profile was generated via simulation of Gaussian random fields using the turning bands method.^42^^–^^44^ The correlation length $l_{c}$ of refractive index fluctuations was based on the analysis presented in Sec. 2.1. The values assigned to R, n, $n_{\text{out}}$, and the extent δn of refractive index fluctuations, on the other hand, were consistent with observations or estimations reported in prior studies.^15^^,^^30^^,^^32^^,^^35^^,^^45^^–^^49^ Table 1 lists all the parameters used to construct spherical nuclear models employed in our work. Note that the default values for R and n used to assess $l_{c}$- or δn-dependent trends, and the default values for $l_{c}$ and δn used to assess R- or n-dependent trends are highlighted in bold.

**Table 1:** Parameters used to construct spherical nuclear models that are optically denser [or less dense] than the embedding cytoplasm. The default values for R and n used to assess $l_{c}$- or δn-dependent trends, and the default values for $l_{c}$ and δn used to assess R- or n-dependent trends are highlighted in bold.

Parameter	Possible values
R (μm)	3.5, **4.0**, 4.5
n	1.39, **1.40**, 1.41 [1.35, **1.36**, 1.37]
$n_{\text{out}}$	1.36 [1.40]
$l_{c}$ (μm)	0.4, 0.5, 0.6, **0.7**, 0.8, 0.9, 1.0
δn	0.005, 0.010, 0.015, **0.020**, 0.025, 0.030, 0.035

#### 2.2.2 Spherical nuclear models that are optically less dense than the embedding cytoplasm

Here, each nuclear model was again constructed as a sphere of radius R but with $n_{\text{out}}>n$. The rest of the construction algorithm was similar to that described above. The entries in square brackets in Table 1 provide the values for n and $n_{\text{out}}$ used to construct these alternative models.

#### 2.2.3 Ellipsoidal nuclear models that are optically denser than the embedding cytoplasm

As shown in Fig. 1, not all cell nuclei can be assumed to be spherical. Hence, the algorithm described above to construct spherical models was also used to construct a set of ellipsoidal models with $n_{\text{out}}<n$. Each model was assigned semiaxis lengths of $S_{x}$, $S_{y}$, and $S_{z}$ corresponding to x, y, and z directions, respectively. Table 2 lists all the relevant parameters employed in construction of these models. As in Table 1, the default values for $S_{x}$, $S_{y}$, $S_{z}$, n, $l_{c}$, and δn are highlighted in bold.

**Table 2:** Parameters used to construct ellipsoidal nuclear models that are optically denser than the embedding cytoplasm. The default values for $S_{x}$, $S_{y}$, $S_{z}$, and n used to assess $l_{c}$- or δn-dependent trends, and the default values for $l_{c}$ and δn used to assess $S_{x}$-, $S_{y}$-, $S_{z}$-, or n-dependent trends are highlighted in bold.

Parameter	Possible values
$S_{x}$ (μm)	2.5, **3.0**, 3.5
$S_{y}$ (μm)	4.5, **5.0**, 5.5
$S_{z}$ (μm)	3.5, **4.0**, 4.5
n	1.39, **1.40**, 1.41
$n_{\mathrm{out}}$	1.36
$l_{c}$ (μm)	0.4, 0.5, 0.6, **0.7**, 0.8, 0.9, 1.0
δn	0.005, 0.010, 0.015, **0.020**, 0.025, 0.030, 0.035

### 2.3 Electromagnetic Simulations

The finite-difference time-domain (FDTD) method is based on a marching-in-time procedure that can be used to simulate propagation of electromagnetic waves through an arbitrarily complex structure constructed in a 3D grid. Extensive details regarding the theoretical basis and implementation of the FDTD method are available in Ref. 50. There are also numerous references that report its application to analysis of optical scattering from tissue constituents.^14^^–^^18^^,^^21^^,^^23^^,^^30^^,^^51^

**Fig. 2:**
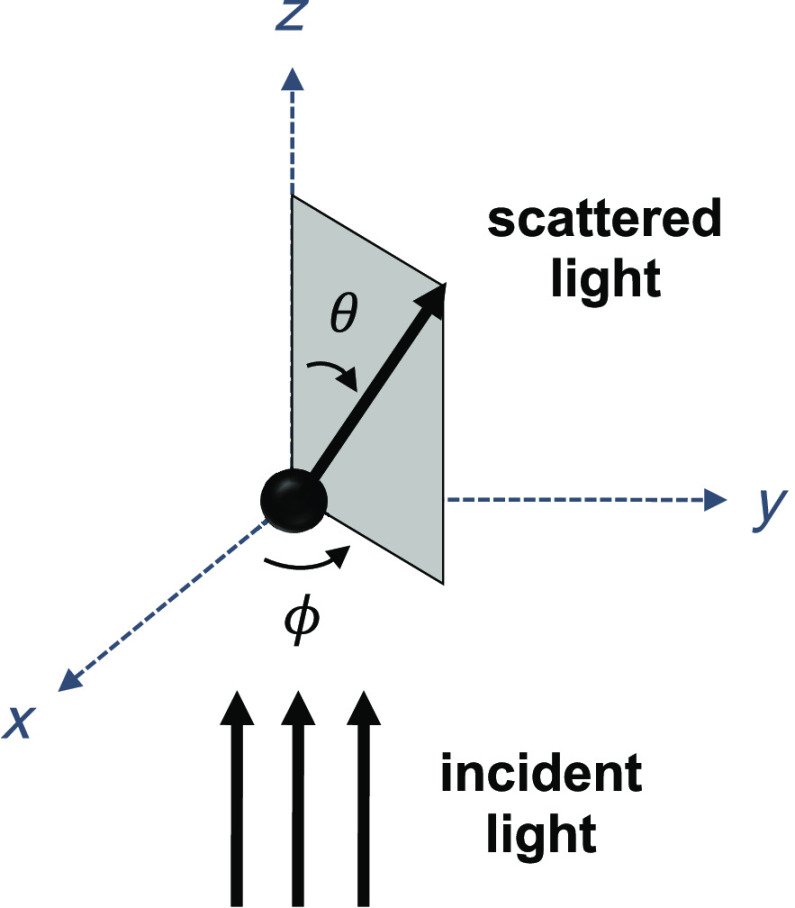
FDTD simulation geometry for an x-polarized incident plane wave propagating in the positive z direction along with a pictorial depiction of polar scattering angle θ and azimuthal scattering angle ϕ.

The FDTD code employed for the study described here has been previously described.^30^^,^^51^ All the simulations were carried out with an 800-nm incident wave propagating in the positive z direction. The electric field was oriented along the x direction, resulting in an x-polarized plane wave. The output of each simulation corresponding to a given nuclear model was a 2D scattering intensity pattern denoted by I(θ,ϕ), where θ∈{0,1,…,180} was the polar scattering angle defined to be the angle between the incident and scattered light directions, and ϕ∈{0,1,…,360} was the azimuthal scattering angle defined to be the angle between the incident wave polarization direction and the scattering plane, both in degrees. Figure 2 provides a pictorial depiction of these angles in connection with our simulation geometry.

### 2.4 Processing of Scattering Patterns and Feature Extraction

We first obtained conventional intensity features corresponding to all the 2D scattering patterns generated. The intensity features considered in this work included mean values of $\log_{10}[I(\theta ,\phi )/I_{o}]$ computed over different angular ranges, where $I_{o}$ was a predefined reference intensity used as a normalization factor. Next, we rescaled each $\log_{10}[I(\theta ,\phi )]$ via a full-scale contrast stretch algorithm to produce image-like functions denoted by $\tilde{I}(\theta ,\phi )$ with values varying between 0 and 255. We created the gray level co-occurrence matrices (GLCMs)^52^^,^^53^ for these functions using eight gray levels and an offset of one along the θ or ϕ direction:

$$G_{\theta }(i,j)=\text{number of pixels with }\lfloor \overset{˜}{I}(\theta ,\phi )/32\rfloor =i\;\text{and}\;\lfloor \overset{˜}{I}(\theta +1,\phi )/32\rfloor =j,\;G_{\phi }(i,j)=\text{number of pixels with }\lfloor \overset{˜}{I}(\theta ,\phi )/32\rfloor =i\;\text{and}\;\lfloor \overset{˜}{I}(\theta ,\phi +1)/32\rfloor =j,$$ (2)

where i,j∈{0,1,…,7}, the (i,j)’th element of $G_{\theta }$ or $G_{\phi }$ represents the frequency of occurrence of two neighboring pixels along the θ or ϕ direction with gray levels i and j, and ⌊·⌋ is the floor function. The GLCMs in Eq. (2) were then normalized to express the matrix elements as probability measures:

$$p_{\theta ,\phi }(i,j)=\frac{G_{\theta ,\phi }(i,j)}{\sum_{i}\sum_{j}G_{\theta ,\phi }(i,j)}.$$ (3)

We used the normalized co-occurrence matrices in Eq. (3) to calculate a series of Haralick features^52^^,^^53^ over different angular ranges. One particular feature considered here was contrast as defined in Eq. (4) below and available in MATLAB’s Image Processing Toolbox:

$${\text{contrast}}_{\theta ,\phi }=\underset{i}{\sum }\underset{j}{\sum }{\left|i-j\right|}^{2}p_{\theta ,\phi }(i,j).$$ (4)

Contrast computed using an offset along the ϕ direction and denoted by ${\text{contrast}}_{\phi }$ is hereafter referred to as azimuthal contrast and is expected to assume large values for frequent and large intensity variations along the ϕ direction. Similarly, contrast computed using an offset along the θ direction and denoted by ${\text{contrast}}_{\theta }$ is hereafter referred to as polar contrast and is expected to assume large values for frequent and large intensity variations along the θ direction. Finally, the ratio of azimuthal contrast to polar contrast is denoted by ${\text{contrast}}_{\phi /\theta }$ and hereafter referred to as contrast ratio.

We analyzed three different angular ranges to determine optimal conditions for detection of changes in subnuclear refractive index fluctuations. The first covers θ=0 deg−40 deg and ϕ=0 deg−360 deg and is indicative of small-angle scattering. The second covers θ=40 deg−140 deg and ϕ=0 deg−360 deg and is the side scattering range. Finally, the third covers θ=140 deg−180 deg and ϕ=0 deg−360 deg and corresponds to high-angle scattering.

It is important to note that three different nuclear models were constructed for each combination of parameters listed in Tables 1 and 2. All the features presented represent averages over these three cases; the standard errors calculated and displayed ensure a proper assessment of the consistency of the results observed for our stochastically inhomogeneous models.

## 3 Results

### 3.1 Nuclear Models

**Fig. 3:**
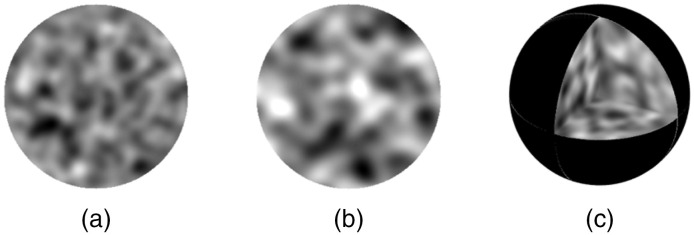
Sample spherical nuclear models of radius R=4.0  μm and mean refractive index n=1.40 embedded in a cytoplasmic medium with a refractive index of $n_{\text{out}}=1.36$: (a) and (b) central cross sections from two different models with $l_{c}=0.5\;\mu m$ and δn=0.010, and $l_{c}=0.7\;\mu m$ and δn=0.020, respectively, and (c) a 3D view of the model shown in (a). The grayscale has been adjusted so that darker areas correspond to regions of higher refractive index.

Figure 3 shows grayscale depiction of sample spherical nuclear models that can be directly fed into the FDTD code. Here, the overall radius and mean refractive index of the models are R=4.0  μm and n=1.40, and these models are embedded in a cytoplasmic medium with a refractive index of $n_{\text{out}}=1.36$. Figures 3(a) and 3(b) represent central cross sections from two different models; in Fig. 3(a), $l_{c}=0.5\;\mu m$ and δn=0.010, whereas in Fig. 3(b), $l_{c}=0.7\;\mu m$ and δn=0.020. Figure 3(c) is a 3D view of the model shown in Fig. 3(a). Note that the grayscale has been adjusted so that darker areas correspond to regions of higher refractive index. It can be seen that our construction algorithm can indeed capture the degree of subnuclear inhomogeneity exemplified by the images shown in Fig. 1.

### 3.2 Two-Dimensional Scattering Patterns

**Fig. 4:**
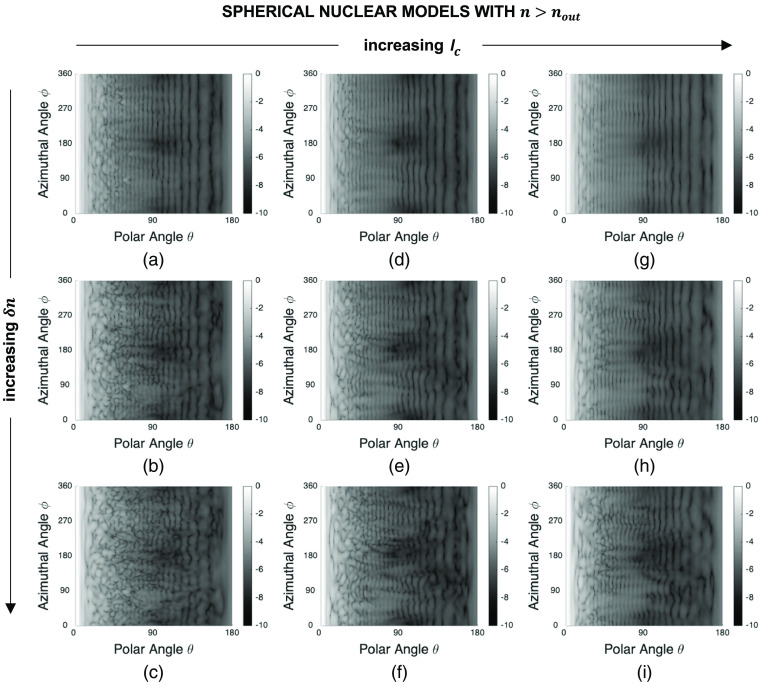
Sample FDTD patterns obtained for spherical nuclear models of radius R=4.0  μm and mean refractive index n=1.40 embedded in a cytoplasmic medium with a refractive index of $n_{\text{out}}=1.36$: (a) $l_{c}=0.5\;\mu m$, δn=0.010, (b) $l_{c}=0.5\;\mu m$, δn=0.020, (c) $l_{c}=0.5\;\mu m$, δn=0.030, (d) $l_{c}=0.7\;\mu m$, δn=0.010, (e) $l_{c}=0.7\;\mu m$, δn=0.020, (f) $l_{c}=0.7\;\mu m$, δn=0.030, (g) $l_{c}=0.9\;\mu m$, δn=0.010, (h) $l_{c}=0.9\;\mu m$, δn=0.020, and (i) $l_{c}=0.9\;\mu m$, δn=0.030. Each pattern has been normalized to a maximum intensity of one and the resulting values have been plotted on a log scale.

Figure 4 shows a set of sample FDTD patterns obtained for spherical models embedded in a cytoplasmic medium with a refractive index of $n_{\text{out}}=1.36$. These patterns demonstrate the influence of changing the correlation length $l_{c}$ and extent δn of nuclear refractive index fluctuations while the overall radius and mean refractive index of the models are kept fixed at their default values of R=4.0  μm and n=1.40. Note that each pattern has been normalized to a maximum intensity of one and the resulting values have been plotted on a log scale. We can see that the intensity of scattered light is highly dependent on the azimuthal angle ϕ for small $l_{c}$ or large δn. As $l_{c}$ increases, the patterns become more regular with less intensity variations along the ϕ direction. As δn increases, on the other hand, the patterns become more irregular with significant intensity variations along the ϕ direction.

**Fig. 5:**
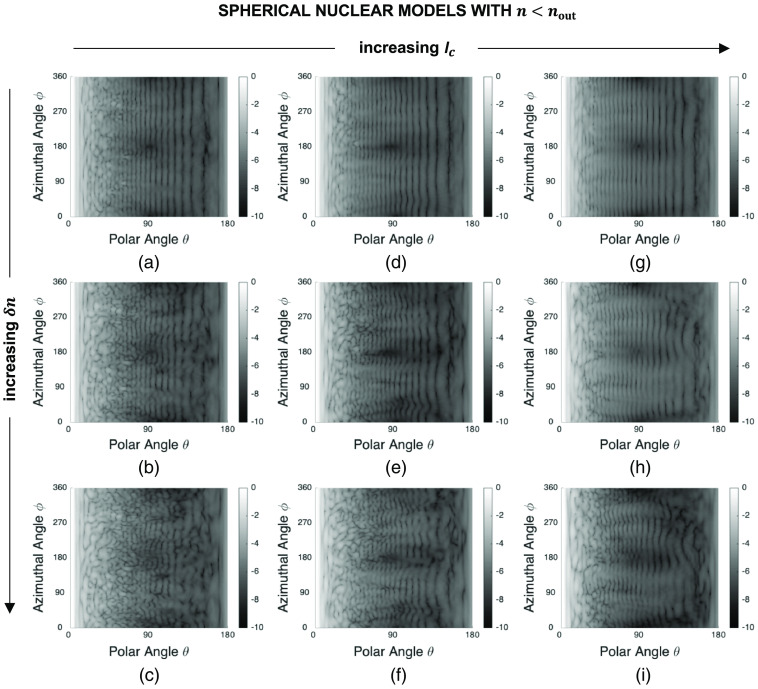
Sample FDTD patterns obtained for spherical nuclear models of radius R=4.0  μm and mean refractive index n=1.36 embedded in a cytoplasmic medium with a refractive index of $n_{\text{out}}=1.40$: (a) $l_{c}=0.5\;\mu m$, δn=0.010, (b) $l_{c}=0.5\;\mu m$, δn=0.020, (c) $l_{c}=0.5\;\mu m$, δn=0.030, (d) $l_{c}=0.7\;\mu m$, δn=0.010, (e) $l_{c}=0.7\;\mu m$, δn=0.020, (f) $l_{c}=0.7\;\mu m$, δn=0.030, (g) $l_{c}=0.9\;\mu m$, δn=0.010, (h) $l_{c}=0.9\;\mu m$, δn=0.020, and (i) $l_{c}=0.9\;\mu m$, δn=0.030. Each pattern has been normalized to a maximum intensity of one and the resulting values have been plotted on a log scale.

The patterns obtained for spherical nuclear models of radius R=4.0  μm and mean refractive index n=1.36 embedded in a cytoplasmic medium with a refractive index of $n_{\text{out}}=1.40$ are shown in Fig. 5. These patterns are comparable to those presented in Fig. 4; all the characteristics noted above for spherical nuclear models that are optically denser than the embedding cytoplasm are also observed for models that are optically less dense than the embedding cytoplasm.

**Fig. 6:**
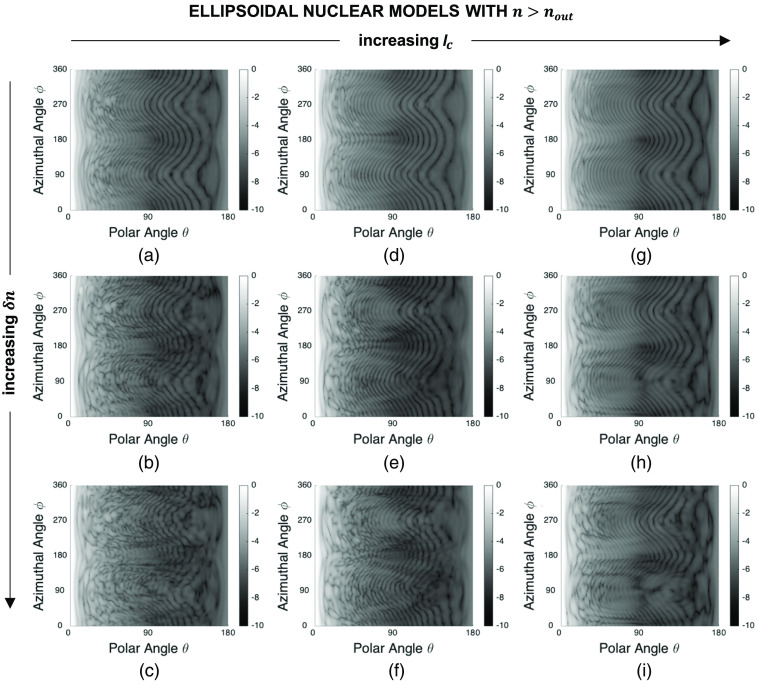
Sample FDTD patterns obtained for ellipsoidal nuclear models of semiaxis lengths $S_{x}=3.0\;\mu m$, $S_{y}=5.0\;\mu m$, and $S_{z}=4.0\;\mu m$, and mean refractive index n=1.40 embedded in a cytoplasmic medium with a refractive index of $n_{\text{out}}=1.36$: (a) $l_{c}=0.5\;\mu m$, δn=0.010, (b) $l_{c}=0.5\;\mu m$, δn=0.020, (c) $l_{c}=0.5\;\mu m$, δn=0.030, (d) $l_{c}=0.7\;\mu m$, δn=0.010, (e) $l_{c}=0.7\;\mu m$, δn=0.020, (f) $l_{c}=0.7\;\mu m$, δn=0.030, (g) $l_{c}=0.9\;\mu m$, δn=0.010, (h) $l_{c}=0.9\;\mu m$, δn=0.020, and (i) $l_{c}=0.9\;\mu m$, δn=0.030. Each pattern has been normalized to a maximum intensity of one and the resulting values have been plotted on a log scale.

Figure 6 shows a set of sample patterns obtained for ellipsoidal nuclear models of semiaxis lengths $S_{x}=3.0\;\mu m$, $S_{y}=5.0\;\mu m$, and $S_{z}=4.0\;\mu m$, and mean refractive index n=1.40 embedded in a cytoplasmic medium with a refractive index of $n_{\text{out}}=1.36$. Vertical background fringes characterizing the patterns in Figs. 4 and 5 are no longer observed for ellipsoidal models which rather exhibit curved fringes. It is important to note, however, that, $l_{c}$- or δn-dependent trends are comparable; the patterns become more irregular with decreasing $l_{c}$ or increasing δn.

### 3.3 Intensity and Contrast Features

**Fig. 7:**
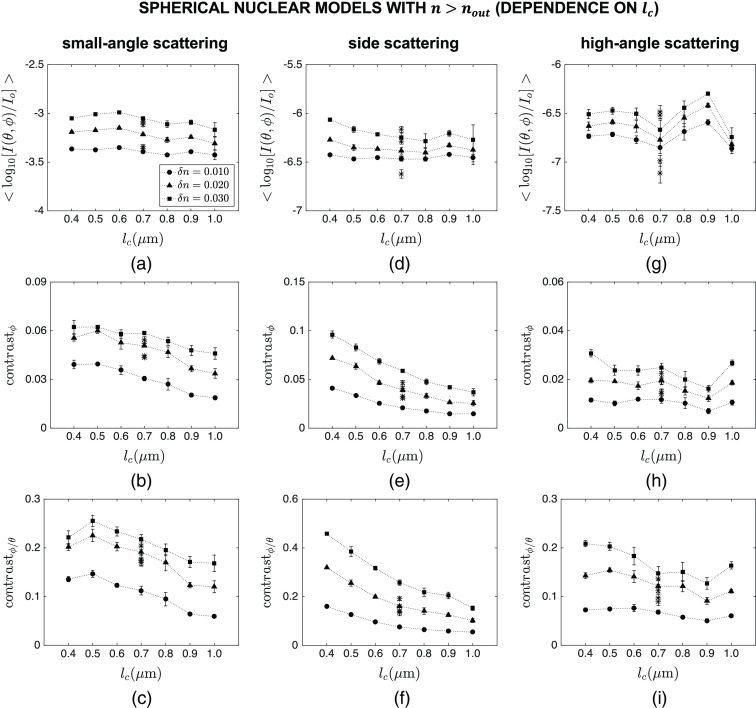
Dependence of mean intensity $\left\langle \log_{10}[I(\theta ,\phi )/I_{o}]\right\rangle$, azimuthal contrast ${\text{contrast}}_{\phi }$, and contrast ratio ${\text{contrast}}_{\phi /\theta }$ on $l_{c}$ for spherical nuclear models that are optically denser than the embedding cytoplasm. Three different angular ranges considered are: (a)–(c) θ=0 deg−40 deg and ϕ=0 deg−360 deg, (d)–(f) θ=40 deg−140 deg and ϕ=0 deg−360 deg, and (g)–(i) θ=140 deg−180 deg and ϕ=0 deg−360 deg. The main markers in these figures correspond to default values of R=4.0  μm and n=1.40; the influence of varying R and n over the ranges given in Table 1 is displayed with asterisk markers for the specific combination of $l_{c}=0.7\;\mu m$ and δn=0.020.

**Fig. 8:**
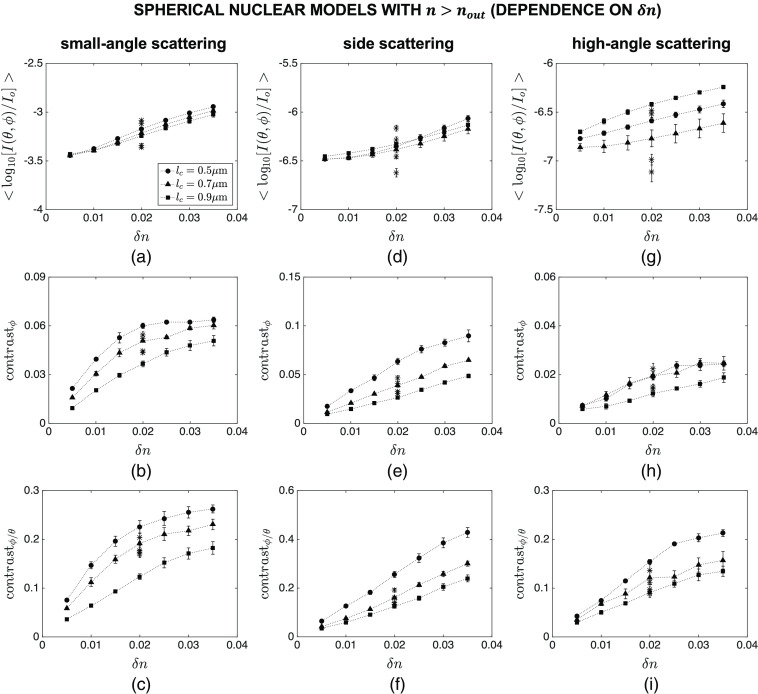
Dependence of mean intensity $\left\langle \log_{10}[I(\theta ,\phi )/I_{o}]\right\rangle$, azimuthal contrast ${\text{contrast}}_{\phi }$, and contrast ratio ${\text{contrast}}_{\phi /\theta }$ on δn for spherical nuclear models that are optically denser than the embedding cytoplasm. Three different angular ranges considered are: (a)–(c) θ=0 deg−40 deg and ϕ=0 deg−360 deg, (d)–(f) θ=40 deg−140 deg and ϕ=0 deg−360 deg, and (g)–(i) θ=140 deg−180 deg and ϕ=0 deg−360 deg. The main markers in these figures correspond to default values of R=4.0  μm and n=1.40; the influence of varying R and n over the ranges given in Table 1 is displayed with asterisk markers for the specific combination of $l_{c}=0.7\;\mu m$ and δn=0.020.

Figures 7 and 8 show the intensity and contrast features computed over different angular ranges for spherical nuclear models that are optically denser than the embedding cytoplasm. The results showing the dependence of mean intensity $\left\langle \log_{10}[I(\theta ,\phi )/I_{o}]\right\rangle$, azimuthal contrast ${\text{contrast}}_{\phi }$, and contrast ratio ${\text{contrast}}_{\phi /\theta }$ on $l_{c}$ are plotted in Fig. 7, whereas the results showing the dependence of the same features on δn are plotted in Fig. 8. Note that the main markers in these figures correspond to default values of R=4.0  μm and n=1.40; the influence of varying R and n over the ranges given in Table 1 is displayed with asterisk markers for the specific combination of $l_{c}=0.7\;\mu m$ and δn=0.020. Although mean intensity does not exhibit a particularly meaningful trend with increasing $l_{c}$ [Figs. 7(a)–7(c)], it appears to increase monotonically with increasing δn for all the angular ranges considered [Figs. 8(a)–8(c)]. It is obvious, however, that changes in the overall radius R and mean refractive index n of the constructed nuclear models lead to more extensive intensity changes that can easily mask observation of subnuclear refractive index variations. The results also illustrate that azimuthal contrast computed over the specific angular range of θ=40 deg−140 deg and ϕ=0 deg−360 deg is highly sensitive to both $l_{c}$ and δn, decreasing monotonically with increasing $l_{c}$ [Fig. 7(e)] and increasing monotonically with increasing δn [Fig. 8(e)]; more importantly, this feature appears to be rather insensitive to changes in R and n, thereby allowing for selective tracking of changes in subnuclear refractive index variations. It is also important to point out that azimuthal contrast computed over θ=0 deg−40 deg and ϕ=0 deg−360 deg or θ=140 deg−180 deg and ϕ=0 deg−360 deg is not characterized by such a consistent trend. Similar comments can be made with regard to the results obtained for contrast ratio; when computed over θ=40 deg−140 deg and ϕ=0 deg−360 deg, this feature is highly sensitive to subnuclear refractive index profile and is only minimally affected by changes in R and n [Figs. 7(f) and 8(f)].

**Fig. 9:**
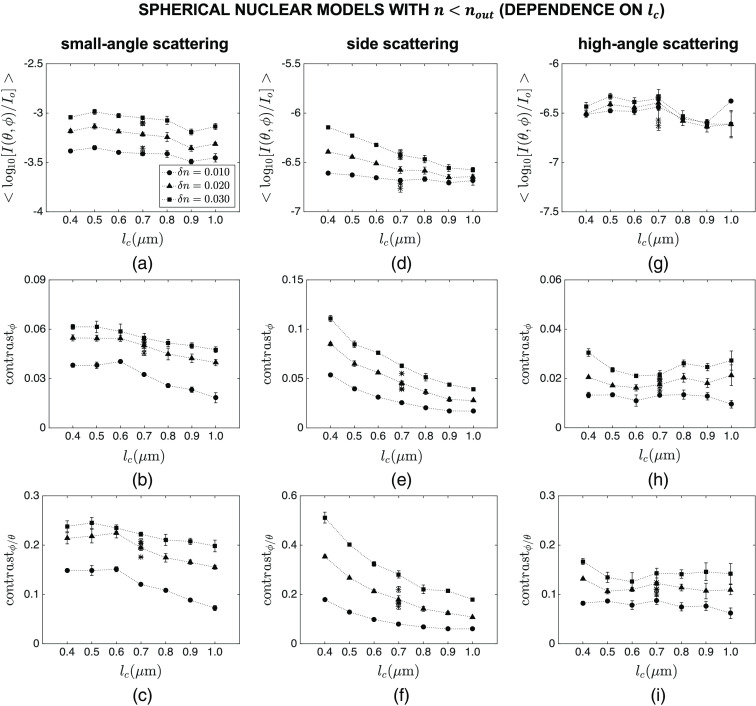
Dependence of mean intensity $\left\langle \log_{10}[I(\theta ,\phi )/I_{o}]\right\rangle$, azimuthal contrast ${\text{contrast}}_{\phi }$, and contrast ratio ${\text{contrast}}_{\phi /\theta }$ on $l_{c}$ for spherical nuclear models that are optically less dense than the embedding cytoplasm. Three different angular ranges considered are: (a)–(c) θ=0 deg−40 deg and ϕ=0 deg−360 deg, (d)–(f) θ=40 deg−140 deg and ϕ=0 deg−360 deg, and (g)–(i) θ=140 deg−180 deg and ϕ=0 deg−360 deg. The main markers in these figures correspond to default values of R=4.0  μm and n=1.36; the influence of varying R and n over the ranges given in Table 1 is displayed with asterisk markers for the specific combination of $l_{c}=0.7\;\mu m$ and δn=0.020.

**Fig. 10:**
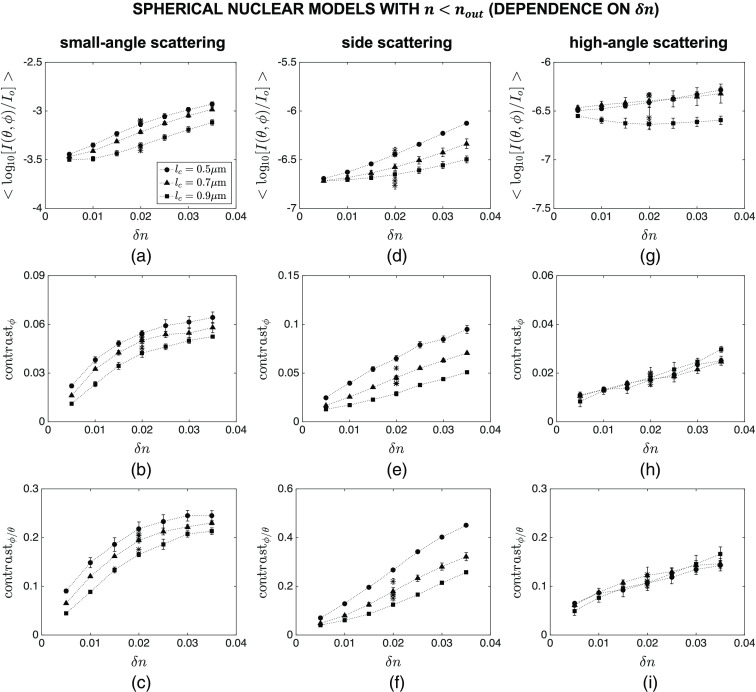
Dependence of mean intensity $\left\langle \log_{10}[I(\theta ,\phi )/I_{o}]\right\rangle$, azimuthal contrast ${\text{contrast}}_{\phi }$, and contrast ratio ${\text{contrast}}_{\phi /\theta }$ on δn for spherical nuclear models that are optically less dense than the embedding cytoplasm. Three different angular ranges considered are: (a)–(c) θ=0  deg−40  deg and ϕ=0  deg−360  deg, (d)–(f) θ=40  deg−140  deg and ϕ=0  deg−360  deg, and (g)–(i) θ=140  deg−180  deg and ϕ=0  deg−360  deg. The main markers in these figures correspond to default values of R=4.0  μm and n=1.36; the influence of varying R and n over the ranges given in Table 1 is displayed with asterisk markers for the specific combination of $l_{c}=0.7\;\mu m$ and δn=0.020.

The intensity and contrast features computed for spherical nuclear models that are optically less dense than the embedding cytoplasm are shown in Figs. 9 and 10. The results are mostly similar to those observed for optically dense spherical models. Mean intensity computed for all the angular ranges considered is highly dependent on R and n, pointing to the fact that this feature cannot be used to track changes in $l_{c}$ and δn. Azimuthal contrast and contrast ratio computed over θ=40 deg−140 deg and ϕ=0 deg−360 deg are both sensitive to $l_{c}$ and δn, decreasing monotonically with increasing $l_{c}$ [Figs. 9(e) and 9(f)] and increasing monotonically with increasing δn [Figs. 10(e) and 10(f)]; further, changes in R and n do not have an overwhelming influence on these features.

**Fig. 11:**
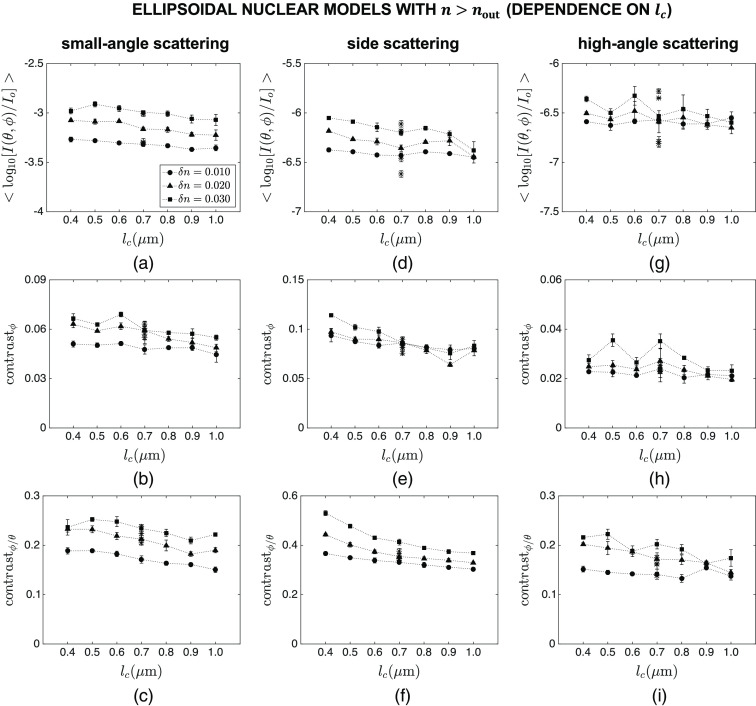
Dependence of mean intensity $\left\langle \log_{10}[I(\theta ,\phi )/I_{o}]\right\rangle$, azimuthal contrast ${\text{contrast}}_{\phi }$, and contrast ratio ${\text{contrast}}_{\phi /\theta }$ on $l_{c}$ for ellipsoidal nuclear models that are optically denser than the embedding cytoplasm. Three different angular ranges considered are: (a)–(c) θ=0 deg−40 deg and ϕ=0 deg−360 deg, (d)–(f) θ=40 deg−140 deg and ϕ=0 deg−360 deg, and (g)–(i) θ=140 deg−180 deg and ϕ=0 deg−360 deg. The main markers in these figures correspond to default values of $S_{x}=3.0\;\mu m$, $S_{y}=5.0\;\mu m$, $S_{z}=4.0\;\mu m$, and n=1.40; the influence of varying $S_{x}$, $S_{y}$, $S_{z}$, and n over the ranges given in Table 2 is displayed with asterisk markers for the specific combination of $l_{c}=0.7\;\mu m$ and δn=0.020.

**Fig. 12:**
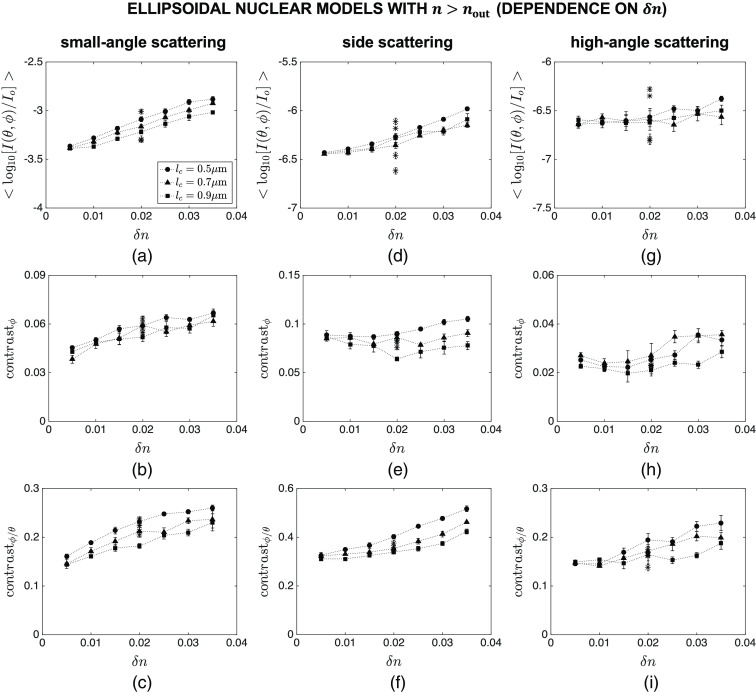
Dependence of mean intensity $\left\langle \log_{10}[I(\theta ,\phi )/I_{o}]\right\rangle$, azimuthal contrast ${\text{contrast}}_{\phi }$, and contrast ratio ${\text{contrast}}_{\phi /\theta }$ on δn for ellipsoidal nuclear models that are optically denser than the embedding cytoplasm. Three different angular ranges considered are: (a)–(c) θ=0 deg−40 deg and ϕ=0 deg−360 deg, (d)–(f) θ=40 deg−140 deg and ϕ=0 deg−360 deg, and (g)–(i) θ=140 deg−180 deg and ϕ=0 deg−360 deg. The main markers in these figures correspond to default values of $S_{x}=3.0\;\mu m$, $S_{y}=5.0\;\mu m$, $S_{z}=4.0\;\mu m$, and n=1.40; the influence of varying $S_{x}$, $S_{y}$, $S_{z}$, and n over the ranges given in Table 2 is displayed with asterisk markers for the specific combination of $l_{c}=0.7\;\mu m$ and δn=0.020.

Finally, Figs. 11 and 12 show the intensity and contrast features computed for ellipsoidal nuclear models that are optically denser than the embedding cytoplasm. The main markers in these figures correspond to default values of $S_{x}=3.0\;\mu m$, $S_{y}=5.0\;\mu m$, $S_{z}=4.0\;\mu m$, and n=1.40; the influence of varying $S_{x}$, $S_{y}$, $S_{z}$, and n over the ranges given in Table 2 is displayed with asterisk markers for the specific combination of $l_{c}=0.7\;\mu m$ and δn=0.020. The trends observed for mean intensity are similar to those observed for spherical models. Azimuthal contrast, on the other hand, exhibits a different behavior as it does not appear to be as sensitive to $l_{c}$ and δn as expected [Figs. 11(e) and 12(e)]. Interestingly, contrast ratio maintains a high level of sensitivity to subnuclear refractive index variations in ellipsoidal models as well; this is particularly evident for the angular range of θ=40 deg−140 deg and ϕ=0 deg−360 deg, which is characterized by a monotonic decrease with increasing $l_{c}$, a monotonic increase with increasing δn, and a minimal influence of changes in $S_{x}$, $S_{y}$, $S_{z}$, and n [Figs. 11(f) and 12(f)].

## 4 Discussion

This study was aimed at evaluating the potential of optical scattering measurements to provide information on internal refractive index profile of cell nuclei. Our simulation results offer strong evidence for the possibility of identifying scattering features that can be linked to subnuclear refractive index fluctuations.

In constructing nuclear models, we adopted a stochastic approach to mimic refractive index variations that typify subnuclear structure in cervical cells. It is important to point out that different correlation functions can be used to describe the spatial profile of refractive index fluctuations in tissues or tissue components. In particular, the Whittle–Matérn correlation family has been extensively used in a considerable number of studies^9^^,^^11^^,^^34^^–^^37^ to analyze scattering in tissues that are modeled as continuous random media; this family encompasses Gaussian, exponential, power-law, and many other functions as special cases. In our work, we opted for Gaussian functions as in Refs. 31–33 due to their mathematical simplicity and easy interpretability. It is obvious, however, that these functions well represent optical density variations characterizing quantitative histopathology images, giving rise to excellent fits as shown in Fig. 1.

We note that our image analysis routine was mainly targeted at establishing a realistic range for typical length scales of subnuclear refractive index fluctuations. When we applied this routine to the image set at hand, we observed that the relative standard deviation of individual correlation lengths extracted from different rows and columns belonging to a given ROI averaged to ∼5%, and the relative standard deviation of individual correlation lengths extracted from different ROIs belonging to a given nuclear image averaged to another ∼5%. This indicates a high degree of consistency and suggests that correlation length $l_{c}$ as quantified here is indeed a suitable parameter for describing the spatial distribution of refractive index variations. In fact, our established range of 0.4 to 1.0  μm is mostly in line with expectations reported in Refs. 11, 34–36, 54 and is appropriate for revealing how length scale variations associated with reorganization at the bulk chromatin level influence scattering signals. On another note, CIN nuclei in our image set appeared to have larger $l_{c}$ on average when compared to their negative counterparts. We point out, however, that the study presented here was focused on a systematic investigation into how sensitive optical scattering signals are to relevant length scale variations in epithelial cell nuclei. The number of images we analyzed was not sufficient to determine whether there existed significant differences among different diagnostic categories; a detailed statistical analysis will only be possible with an extended image set.

A number of prior studies^10^^,^^55^^–^^59^ tend to characterize internal refractive index profiles of nuclei in terms of a lumped quantity that is defined to be proportional to correlation length $l_{c}$ and extent δn of refractive index fluctuations. This quantity, often referred to as the disorder strength, is observed to increase with progression of cancer; increased disorder strength most likely corresponds to chromatin compaction that is expected to manifest as an increase in $l_{c}$, δn, or both. These observations underlie the necessity to analyze δn-dependent changes in scattering signals in addition to $l_{c}$-dependent changes. Our selected values for δn cover a sufficiently large range to account for possible variations that can accompany cancer development.

We considered three different categories of nuclear models: spherical nuclei that were optically denser than the embedding cytoplasm, spherical nuclei that were optically less dense than the embedding cytoplasm, and ellipsoidal nuclei that were optically denser than the embedding cytoplasm. In all cases, the cytoplasm was assumed to be a homogeneous medium with a fixed refractive index. This is a valid assumption for epithelial cells that are characterized by a very low volume fraction of organelles. The features computed for the first category and presented in Figs. 7 and 8 clearly indicate that azimuthal contrast ${\text{contrast}}_{\phi }$ computed for side scattering exhibits an appreciable sensitivity to correlation length $l_{c}$ and extent δn of subnuclear refractive index fluctuations and is minimally influenced by changes in overall radius R and mean refractive index n of nuclei. Standard error bars are mostly observed to be extremely small, providing evidence for consistency of the trends observed. It is well established that intensity variations along the polar direction are directly related to the overall size and mean refractive index of scattering structures. As such, polar contrast ${\text{contrast}}_{\theta }$ computed over any angular range is not expected to be indicative of either $l_{c}$ or δn. Our simulation results corroborate this expectation; plots of ${\text{contrast}}_{\theta }$ as a function of $l_{c}$ or δn, however, are not included here for brevity. Yet, normalization of ${\text{contrast}}_{\phi }$ by ${\text{contrast}}_{\theta }$ gives rise to contrast ratio ${\text{contrast}}_{\phi /\theta }$ which also proves to be highly sensitive to both $l_{c}$ and δn when computed for side scattering; as evidenced by tight clustering of results corresponding to different R and n, this feature can be quite effective in monitoring the internal refractive index profile of nuclei independently of their overall size and mean refractive index.

Several studies^60^^,^^61^ have recently challenged the long-held perception of cell nuclei as dense structures with mean refractive indices higher than that of the surrounding cytoplasm. These studies led us to construct the second category of nuclear models that are optically less dense than the embedding cytoplasm with the ultimate goal of elucidating any possible effect of such a refractive index inversion. The results in Figs. 9 and 10 illustrate that optical scattering trends remain the same as before; both ${\text{contrast}}_{\phi }$ and ${\text{contrast}}_{\phi /\theta }$ computed for side scattering retain their sensitivity to $l_{c}$ and δn, allowing for assessment of internal refractive index fluctuations. Even though mean nuclear refractive index can be higher or lower than that of the cytoplasm depending on the particular cell type under consideration, it is apparent that our inferences are valid for either case.

Significant differences are known to exist between 2D scattering patterns of spherical and nonspherical structures. It was hence necessary to extend our analysis to ellipsoidal nuclei and determine how effectively our proposed features can capture changes in $l_{c}$ and δn for ellipsoidal models. The results obtained for this third category and presented in Figs. 11 and 12 indicate that azimuthal contrast ${\text{contrast}}_{\phi }$ computed for the side scattering range does not exhibit the desired monotonic dependence on either $l_{c}$ or δn. The underlying reason can easily be traced to respective FDTD patterns exemplified in Fig. 6; intensity variations along the ϕ direction are no longer solely due to internal heterogeneities but to overall nuclear morphology as well. Contrast ratio ${\mathrm{contrast}}_{\phi /\theta }$, on the other hand, intrinsically corrects for intensity variations due to overall morphology, unraveling variations that are directly linked to internal heterogeneities. The resulting monotonic dependence on $l_{c}$ and δn suggests that ${\text{contrast}}_{\phi /\theta }$ for side scattering qualifies as an indicator of changes in subnuclear refractive index fluctuations; small standard error bars again point to consistency of the trends observed. We remark that we did not carry out simulations for ellipsoidal nuclei that were optically less dense than the embedding cytoplasm. Simulations for spherical models, however, provide sufficient evidence that we would essentially make the same inferences about the intensity and contrast features extracted from their scattering patterns.

Taken altogether, our results demonstrate that analysis of azimuthal dependence in 2D scattering patterns of cell nuclei is essential for assessment of internal refractive index fluctuations. It is also especially evident that azimuthal intensity variations are most prominent over the side scattering range. Contrast ratio ${\text{contrast}}_{\phi /\theta }$ computed for side scattering is highly sensitive to $l_{c}$ and δn for all three types of nuclear models considered. More importantly, this feature is not susceptible to changes in the overall size and mean refractive index of nuclei, thereby allowing for selective tracking of changes in subnuclear refractive index fluctuations. In the context of this study, these findings implicate the possibility of selectively monitoring precancer-related alterations in chromatin organization.

Contrary to the notion that high-angle scattering signals can be used to explore internal refractive index profiles of tissue constituents,^14^^–^^16^^,^^62^ our observations reveal that analysis of scattered light intensities over this angular range cannot provide an exclusive insight into refractive index variations due to the dominating influence of overall morphological factors. Further, weak high-angle scattering signals are extremely prone to noise. Thus, shifting the range of interest toward side scattering angles in line with the results presented here is likely to have added benefits. Relatedly, we note that interpretation of intensity features requires a proper calibration so that different measurements can be directly compared. Contrast features, on the other hand, are computed based on self-normalized intensity values, and there is no need for calibration across measurements. These considerations render our analysis methodology highly advantageous from a practical perspective. That said, we would like to point out that the level of grayscale quantization and the offset used to calculate contrast features need to be selected carefully to well characterize intensity variations along the azimuthal or polar directions. Here, quantization to eight gray levels and an offset of one have generated satisfactory and consistent results for our patterns with an angular sampling interval of 1 deg in both directions. These analysis parameters can also be tailored to effectively minimize any influence of noise that will inevitably be present in real measurements regardless of the specific angular range under consideration. In fact, such an optimization process should be an integral part of any study that involves calculation of Haralick features.

To open the way for further investigations, it is also necessary to discuss a few limitations of using quantitative histopathology images to extract $l_{c}$. First, DNA-specific staining raises the issue of whether we can account for the presence of nuclear proteins. In our analysis, we made the simplifying assumption that since DNA and proteins associate closely, these images can indeed provide a realistic map of chromatin organization; this assumption remains to be validated through alternative staining or labeling techniques. Second, the diffraction-limited lateral resolution of the imaging system enables quantification of subnuclear structure down to a scale of 0.34  μm. Yet, some studies^10^^,^^55^^–^^58^ point to the importance of analyzing nuclear changes at subdiffractional length scales. High-resolution imaging techniques can possibly be employed to reveal small-scale structural details which may even entail the use of different correlation functions to better mimic the underlying stochastic profile of cell nuclei. Needless to say, azimuth-dependent intensity variations in scattered light intensity are expected to be sensitive to refractive index fluctuations at length scales smaller than what we considered here, especially when smaller wavelengths are used. Our methodology based on analysis of 2D scattering signals will thus still be potentially applicable for monitoring such fluctuations.

Although the contrast features we propose can be used to assess subnuclear structure, they cannot isolate the effects of $l_{c}$ and δn. An important question that arises is whether it is possible to identify scattering signatures that can be used to independently monitor changes in these parameters. Distinct features that are sensitive only to $l_{c}$ or δn and can hence provide mutually exclusive information on the length scale and extent of refractive index fluctuations will potentially bring out more specific details regarding the internal structure of cell nuclei. We intend to carry out further simulations and tackle this challenging issue as part of our future research efforts.

Technological advances over the past decades have led to a tremendous expansion of high-performance computational resources. However, as pointed out in a recent review article,^63^ this has not been paralleled by an equally noteworthy increase in the number of numerical light scattering studies based on computationally intensive finite-element or finite-difference solutions. One major contributing factor is perhaps the difficulty associated with construction of realistic models; availability of detailed quantitative data is necessary to generate input relevant for cell or tissue analysis. Yet, our work reveals that optical scattering signals contain a wealth of information, signifying a further need for carefully designed and systematic numerical studies to uncover intricate details of biological significance. It is only through a synergistic framework of numerical, analytical, and experimental approaches that the diagnostic potential of scattering measurements can be delineated and fully exploited.

## 5 Conclusions

In summary, the research described here highlights the wide extent of diagnostic information that can be extracted from 2D optical scattering patterns of cell nuclei. Conventional intensity features are mostly dictated by the overall morphology and mean refractive index of nuclei, making it very difficult to exclusively sense subnuclear refractive index fluctuations. Our in-depth analysis on azimuth-dependent variations in scattered light intensity forms the basis of a methodology for monitoring these fluctuations that can provide significant insights into nuclear chromatin organization. Application of the same methodology to track internal refractive index profiles of other subcellular organelles or tissue constituents is likely to pave the way for optical detection of a broad range of disease-related abnormalities.
